# Deciphering the Oncogenic Landscape of Hepatocytes Through Integrated Single‐Nucleus and Bulk RNA‐Seq of Hepatocellular Carcinoma

**DOI:** 10.1002/advs.202412944

**Published:** 2025-02-17

**Authors:** Huanhou Su, Xuewen Zhou, Guanchuan Lin, Chaochao Luo, Wei Meng, Cui Lv, Yuting Chen, Zebin Wen, Xu Li, Yongzhang Wu, Changtai Xiao, Jian Yang, Jiameng Lu, Xingguang Luo, Yan Chen, Paul KH Tam, Chuanjiang Li, Haitao Sun, Xinghua Pan

**Affiliations:** ^1^ Department of Biochemistry and Molecular Biology School of Basic Medical Sciences Southern Medical University and Guangdong Provincial Key Laboratory of Single Cell Technology and Application Guangzhou 510515 China; ^2^ Precision Regenerative Medicine Research Centre Medical Science Division and State Key Laboratory of Quality Research in Chinese Medicine Macau University of Science and Technology Macao 999078 China; ^3^ College of Life Sciences Shihezi University Shihezi Xinjiang 832003 China; ^4^ Clinical Biobank Center Microbiome Medicine Center Department of Laboratory Medicine Guangdong Provincial Clinical Research Center for Laboratory Medicine Zhujiang Hospital Southern Medical University Guangzhou 510280 China; ^5^ Department of Hepatobiliary Surgery I General Surgery Center and Guangdong Provincial Clinical and Engineering Center of Digital Medicine Zhujiang Hospital Southern Medical University Guangzhou 510280 China; ^6^ Department of Psychiatry Yale University School of Medicine New Haven CT 06510 USA; ^7^ Division of Hepatobiliopancreatic Surgery Department of General Surgery Nanfang Hospital Southern Medical University Guangzhou Guangdong 510515 China; ^8^ Key Laboratory of Infectious Diseases Research in South China (China Ministry Education) Southern Medical University Guangzhou Guangdong 510515 China

**Keywords:** hepatocellular carcinoma classification, hepatocyte subtype, metabolic pathway, quantile distribution model, single‐cell transcriptomics

## Abstract

Hepatocellular carcinoma (HCC) is a major cause of cancer‐related mortality, while the hepatocyte mechanisms driving oncogenesis remains poorly understood. In this study, single‐nucleus RNA sequencing of samples from 22 HCC patients revealed 10 distinct hepatocyte subtypes, including beneficial Hep0, predominantly malignant Hep2, and immunosuppressive Hep9. These subtypes were strongly associated with patient prognosis, confirmed in TCGA‐LIHC and Fudan HCC cohorts through hepatocyte composition deconvolution. A quantile‐based scoring method is developed to integrate data from 29 public HCC datasets, creating a Quantile Distribution Model (QDM) with excellent diagnostic accuracy (Area Under the Curve, AUC = 0.968‐0.982). QDM was employed to screen potential biomarkers, revealing that *PDE7B* functions as a key gene whose suppression promotes HCC progression. Guided by the genes specific to Hep0/2/9 subtypes, HCC is categorized into metabolic, inflammatory, and matrix classes, which are distinguishable in gene mutation frequencies, survival times, enriched pathways, and immune infiltration. Meanwhile, the sensitive drugs of the three HCC classes are identified, namely ouabain, teniposide, and TG‐101348. This study presents the largest single‐cell hepatocyte dataset to date, offering transformative insights into hepatocarcinogenesis and a comprehensive framework for advancing HCC diagnostics, prognostics, and personalized treatment strategies.

## Graphical abstract

By combining snRNA‐seq and bulk RNA‐seq, this study identified key hepatocyte subtypes, genes, and pathways in HCC. It established a Quantile Distribution Model, providing potential targets for HCC diagnosis and treatment. It categorized HCC into metabolic, inflammatory, and matrix classes, each with sensitive drugs identified. The findings offer insights into HCC's oncogenic landscape and future applications.

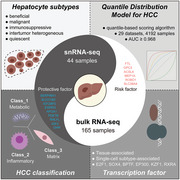

## 1 Introduction

Hepatocellular carcinoma (HCC) is the predominant histological subtype of primary liver cancer, accounting for over 80% of cases, and ranks as the third leading cause of cancer‐related mortality worldwide.^[^ ^1^ ^]^ HCC is a malignant tumor characterized by multi‐gene dysregulation and significant heterogeneity, posing challenges to its diagnosis and therapy. Therefore, precise characterization of the oncogenic mechanisms in HCC is urgently needed in clinical practice.

Traditional bulk RNA sequencing (bRNA‐seq) provides an overall transcriptomic profile of cancer tissues but often obscures gene expression information from rare cell subpopulations. Recently, single‐cell RNA sequencing (scRNA‐seq) has emerged as a powerful tool for exploring liver cancer heterogeneity, particularly in studies of the tumor immune microenvironment and tumor‐associated macrophages and fibroblasts. However, due to their fragility and large size, hepatocytes are often sparsely captured by scRNA‐seq, limiting detailed investigations of hepatocellular carcinogenesis at the single‐cell level. Single‐nucleus RNA sequencing (snRNA‐seq) unbiasedly captures all nuclei within a sample, thereby providing a more accurate representation of the natural cell type composition across various tissues. This method is particularly suited for frozen or challenging‐to‐dissociate samples.^[^ ^2^ ^]^ Consequently, snRNA‐seq enables a comprehensive profiling of hepatocytes beyond immune cells, facilitating more targeted investigations of hepatocytes.^[^ ^3^ ^]^

Serum alpha‐fetoprotein (AFP), despite its widespread use as a pivotal biomarker in HCC diagnosis (specificity 80%–90%), demonstrates limited sensitivity (40%–60%).^[^ ^4^ ^]^ Conversely, multi‐parametric serological models incorporating AFP shows superior diagnostic performance compared to individual biomarkers alone. The GALAD model,^[^ ^5^ ^]^ which utilizes three serological markers (AFP, AFP‐L3, and DCP) along with patient age and sex achieved an area under the receiver operating characteristic curve (AUROC) exceeding 0.90 across multiple international cohorts. In a multi‐institutional cohort study, the GALAD model demonstrated a diagnostic AUROC of 0.935, outperforming individual biomarkers AFP (0.863), AFP‐L3 (0.810), PIVKA‐II (0.872), and the BALAD‐2 model (0.883).^[^ ^6^ ^]^ In recent years, various novel diagnostic models independent of serum protein have emerged, including those driven by DNA methylation (AUROC: 0.978–0.981),^[^ ^7^ ^]^ tumor mutational burden (AUROC: 0.928–0.950),^[^ ^8^ ^]^ and salivary protein glycopatterns (AUROC: 0.857‐0.886).^[^ ^9^ ^]^ While these new models were clinically constrained, they demonstrated significant potential in HCC diagnosis.

The selection of modeling methodologies is a pivotal factor in defining the attributes of HCC diagnostic models, with logistic regression and machine learning algorithms being predominantly utilized. Logistic regression‐based HCC diagnostic models are characterized by simplicity, speed, and strong interpretability, making them well‐suited for linearly separable problems and amenable to facile adjustments. However, their diagnostic performance is relatively constrained (AUROC: 0.862–0.94118)^[^ ^7^, ^10^ ^]^ and they exhibit sensitivity to outliers. Conversely, machine learning‐based HCC diagnostic models demonstrate robust predictive capabilities (AUROC: 0.893–0.99)^[^ ^11^ ^]^ and efficient data handling capacities, adept at managing diverse data types and complex features. However, these models are highly complicated, necessitating specialized technical expertise and high‐performance computing platforms for continual training and refinement, and they frequently lack transparency in their outcomes.

In this study, we applied snRNA‐seq and bRNA‐seq to 77 HCC patients (SMU cohort), conducting a comprehensive analysis of the oncogenic landscape and molecular regulation of HCC. Building on these findings and integrating 29 public human HCC datasets comprising 4192 samples, we developed a quantile‐based scoring method and constructed a Quantile Distribution Model (QDM) for HCC. Additionally, we define three classes of HCC: metabolic‐HCC, inflammatory‐HCC, and matrix‐HCC. We elucidated their clinical characteristics and further identified preferential therapeutic agents for each class of HCC.

## 2 Results

### 2.1 Revealing Major Cell Types and Overall Transcriptomic Profile of HCC Based on snRNA‐Seq

**Figure 1:**
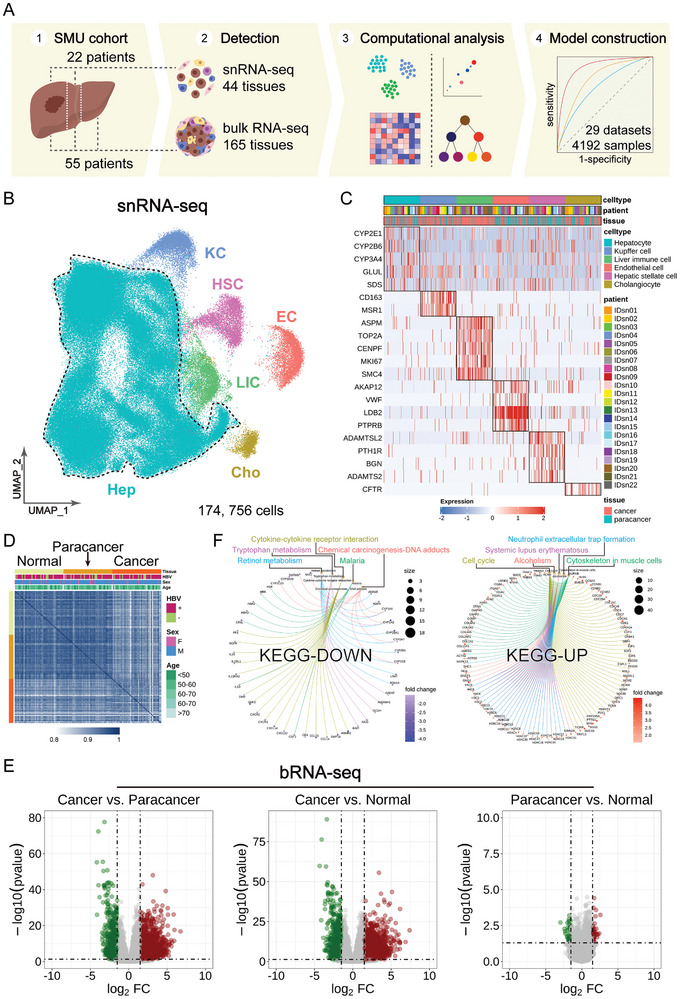
Revealing major cell types and overall transcriptomic profile of HCC based on snRNA‐seq. A) Workflow for detection and analysis of HCC. B) UMAP visualization of major cell types based on snRNA‐seq data, with colors representing different cell types. Samples were obtained from liver tissues of HCC patients undergoing surgical resection, including both cancer and paracancer tissues. C) Heatmap visualization of marker genes for major cell types, with colors representing gene expression levels. D) Sample‐to‐sample correlation across different tissues based on bRNA‐seq data. E) DEGs between different tissues based on bRNA‐seq data, with green indicating down‐regulated genes and red indicating up‐regulated genes. F) KEGG enrichment analysis of co‐downregulated and co‐upregulated DEGs.

To comprehensively understand the cellular composition and diversity of HCC, we collected 209 liver tissue samples from a cohort of 77 HCC patients (SMU cohort, Table S1, Supporting Information), including cancer, paracancer, and normal tissues (**Figure** **1**A). We combined single‐nucleus RNA sequencing (snRNA‐seq; 22 cancer and 22 paracancer samples) with bulk RNA sequencing (bRNA‐seq; 165 samples) to investigate the mechanisms underlying hepatocellular carcinogenesis. Through computational analyses, we identified the major cell types and hepatocyte subtypes in HCC. Based on the characterization of hepatocyte subtypes, we integrated 29 HCC datasets to construct a diagnostic model for HCC.

Using snRNA‐seq data, we obtained single‐cell transcriptomic profiles for 174756 cells and annotated six major cell types using known lineage‐specific marker genes: Hepatocyte (Hep), Liver immune cell (LIC), Endothelial cell (EC), Hepatic stellate cell (HSC), Kupffer cell (KC), and Cholangiocyte (Cho) (Figure 1B,C; Figure S1A‐C, Supporting Information). The proportion of hepatocytes in scRNA‐seq data was ≈ 4.68%–24.48%,^[^ ^12^ ^]^ while it reached 85.23% (148 182/174 756, Table S2, Supporting Information) in our snRNA‐seq data, closely reflecting the liver cellular composition under normal physiological conditions (Figure S1D, Supporting Information).

Using bRNA‐seq data, we conducted a sample‐to‐sample correlation analysis, showing that paracancer and normal tissues share highly similar molecular characteristics compared to cancer tissues (Figure 1D). Differentially expressed genes (DEGs) analysis using thresholds of |log2FoldChange| > 1.5 and *p* < 0.05 (Table S3, Supporting Information) identified 543 downregulated and 1828 upregulated genes in cancer vs paracancer tissues, and 604 downregulated and 2038 upregulated genes in cancer vs normal tissues. Very few DEGs were found between paracancer and normal tissues, underscoring their high similarity (Figure 1E; Figure S1E, Supporting Information). KEGG enrichment analysis of the intersection genes from the cancer vs paracancer group and the cancer vs normal group (Table S4, Supporting Information) revealed that co‐downregulated DEGs were involved in pathways such as “Retinol metabolism, Tryptophan metabolism, Cytokine‐cytokine receptor interaction, Malaria, and Chemical carcinogenesis‐DNA adducts”. Conversely, co‐upregulated DEGs were associated with pathways including “Cell cycle, Systemic lupus erythematosus, Alcoholism, Neutrophil extracellular trap formation, and Cytoskeleton in muscle cells”. Many of these pathways are relevant to HCC and are significant pathogenic factors in its development.^[^ ^13^ ^]^ It is worth noting that *GHR's* role in HCC development is not fully elucidated, however, some studies have shown that *GHR* expression is downregulated in liver cancer tissues.^[^ ^14^ ^]^

### 2.2 Deciphering the Genomic Evolution of Hepatocytes of HCC Regarding its Pathogenesis

**Figure 2:**
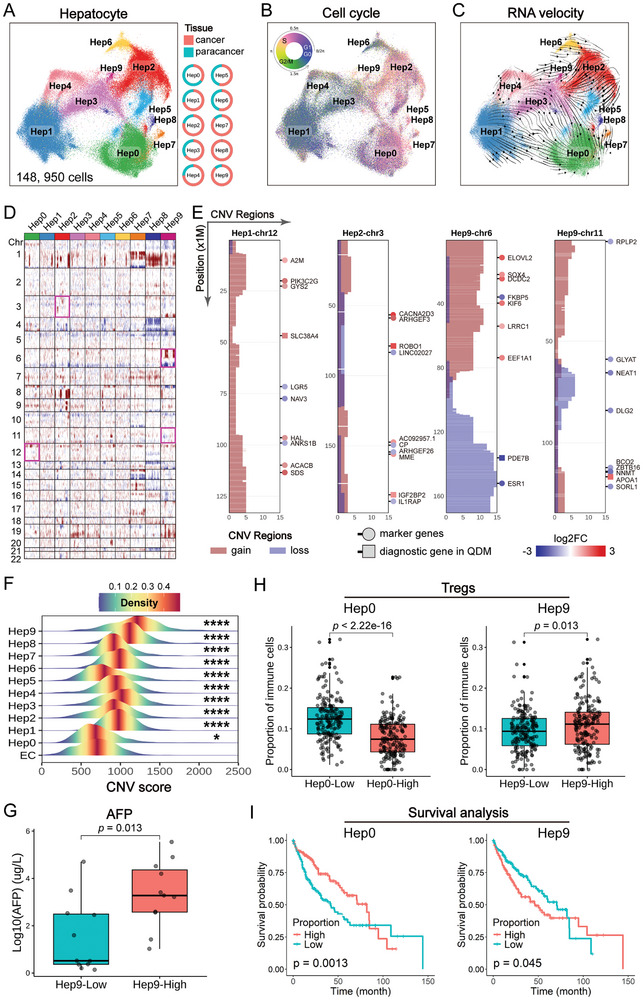
Deciphering the genomic evolution of hepatocytes of HCC regarding its pathogenesis. A) Ten hepatocyte subtypes were identified from the snRNA‐seq data. The circular diagram shows the tissue origin of each hepatocyte subtype. B) Cell cycle status for the hepatocyte subtypes. C) RNA velocity analysis of hepatocyte subtypes. The direction and length of the arrows represent the trend and rate of change in the unspliced‐to‐spliced mRNA ratio. D) Copy number variations (CNVs) in the hepatocyte subtypes. Red indicates copy number gain, and blue indicates copy number loss. E) CNV regions in Hep1‐chr12, Hep2‐chr3, Hep9‐chr6, and Hep9‐chr11. Regions with CNV gains are highlighted in red, and regions with CNV losses are highlighted in blue. Circle symbols represent differential genes, while rectangle symbols denote diagnostic genes from this study; colors indicate fold change differences. F) CNV scores across 10 hepatocyte subtypes relative to endothelial cells. *p < 0.05; ****p < 0.0001. G) Correlation between the proportion of Hep9 in cancer tissues and serum AFP levels of HCC patients. H) Correlation between the proportion of Hep0/9 and the proportion of Tregs in the TCGA cohort. I) Correlation between the proportions of Hep0/9 and patient prognosis in the TCGA cohort.

To elucidate the oncogenic landscape of hepatocytes in HCC, we extracted 148 182 hepatocytes from snRNA‐seq data (**Figure** **2**A), which were reclustered into 10 hepatocyte subtypes (Hep0–Hep9; Table S5, Supporting Information). We examined the tissue origin of these subtypes and found that the majority of cells in Hep7/8/9 originated from cancer tissues (87%, 100%, and 89%, respectively; Table S6, Supporting Information), while most cells in Hep0 originated from paracancer tissues (65%). Further analysis revealed that Hep7 and Hep8 are intertumor heterogeneous subtypes, predominantly derived from individual patients (76% and 87%, respectively; Table S6, Supporting Information). Using the Tricycle^[^ ^15^ ^]^ method based on transfer learning to infer cell cycle stages, we discovered that Hep1 is primarily in the G1/G0 phase, while Hep0/2 are mainly in the S phase (Figure 2B). RNA velocity analysis using scVelo^[^ ^16^ ^]^ revealed that Hep1 exhibits the least active transcriptional dynamics, characterized by the lowest levels of unspliced RNA (Figure 2C). These results suggest that Hep1 represents a quiescent hepatocyte subtype.

We employed inferCNV^[^ ^17^ ^]^ to identify copy number variations (CNVs) across various hepatocyte subtypes, revealing distinct CNV region patterns. For instance, Hep8 exhibits large‐scale CNV gains on chromosome 1 (position: 146.94–235.65 million), while Hep7 shows large‐scale gains (position: 116.37–186.42 million) and losses (position: 226.90–247.17 million) (Figure 2D). These differences in CNV patterns may be associated with cellular pathologic heterogeneity and clonal diversity within HCC. We specifically analyzed CNV patterns in Hep0/2/9 in relation to HCC progression. Examination of CNV regions in Hep1‐chr12, Hep2‐chr3, Hep9‐chr6, and Hep9‐chr11 (Figure 2E) revealed corresponding changes in DEGs (top 10 genes) consistent with CNV patterns, notably the *PDE7B* gene within a high‐frequency loss CNV region on chr6, which is downregulated in Hep9. *PDE7B* was included as a diagnostic gene in subsequent analyses based on systematic criteria.

To explore the genomic evolution pattern, we constructed subclonal architectures for each patient using the inferCNV pipeline and visualized tumor evolution with UPhyloplot2.^[^ ^18^ ^]^ This analysis revealed consistency in early CNV events, including chromosomal segment gains at 1q, 7q, 9q, 11p, 12p, 16p, 19p, and 19q, observed in over 30% of patients (Figure S2, Supporting Information). Notably, a gain in 19q was present in 72.7% (16/22) of patients, highlighting its potential role in HCC onset.

CNV scores showed that Hep0–9 were significantly higher than the reference endothelial cells, with Hep9 ranking at the top (Figure 2F). We further examined the clinical relevance of Hep9 in the 22 snRNA‐seq HCC cases from the SMU cohort. Patients were categorized into Hep9‐High and Hep9‐Low groups according to the proportion of Hep9 in their cancer tissues. The results revealed that the serum AFP levels were significantly higher in the Hep9‐High group compared to the Hep9‐Low group (*p* = 0.013, Figure 2G). Using CIBERSORTx,^[^ ^19^ ^]^ we decomposed 22 immune cell types from the TCGA‐LIHC cohort^[^ ^20^ ^]^ and found that HCC tissues with a high proportion of Hep9 had increased levels of regulatory T cells (Tregs) (*p* = 0.013, Figure 2H), which inhibit cancer immune surveillance and promoted tumor development.^[^ ^13^ ^]^ Conversely, a lower proportion of Hep0 was associated with a higher proportion of Tregs (*p* < 0.0001). Importantly, leveraging the characteristic spectra of hepatocyte subtypes identified in snRNA‐seq, we decomposed the cellular composition of the TCGA cohort and found that a high proportion of Hep9 (*p* = 0.045) and a low proportion of Hep0 (*p* = 0.0013) were associated with poor prognosis in HCC (Figure 2I). Finally, we identified Hep9 as an immunosuppressive hepatocyte subtype, Hep0 as a beneficial hepatocyte subtype, Hep1 as a quiescent subtype, Hep2 as a predominantly malignant subtype, Hep3–6 as transitional phase hepatocyte subtypes, and Hep7–8 as intertumor heterogeneous subtypes.

### 2.3 Decoding Molecular Pathways of Hepatocytes of HCC with snRNA‐Seq

Using Hep0 as the reference, we performed multi‐pathway enrichment analysis^[^ ^21^ ^]^ to identify pathways with downregulated gene expression across Hep1 to Hep9. Results showed a generalized suppression of hepatic metabolic functions, particularly in energy metabolism and amino acid metabolism pathways, including “Pyruvate metabolism, Butanoate metabolism, Fatty acid degradation, and Tryptophan metabolism” (Figure S3A, Supporting Information). Additionally, downregulation of “ABC transporters” and the “PPAR signaling pathway” was observed in multiple hepatocyte subtypes, suggesting their important role in HCC progression, particularly in cases associated with “Alcoholic liver disease”.^[^ ^22^ ^]^

**Figure 3:**
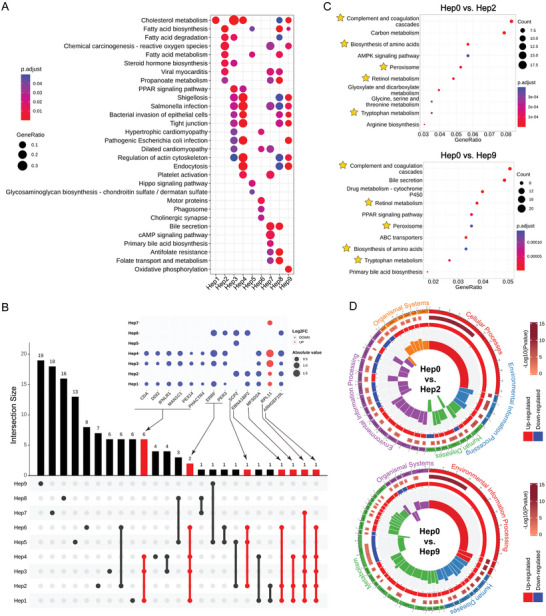
Decoding molecular pathways of hepatocytes of HCC with snRNA‐seq. A) Multi‐pathway enrichment analysis of upregulated genes in Hep1‐9. B) Upset plot of the top 20 DEGs in Hep1‐9. The bubble plot in the upper‐right corner shows the expression levels of these genes across the corresponding hepatocyte subtypes. C) KEGG analysis of marker genes in the Hep0 group, using Hep2 and Hep9 as controls (top/bottom). * indicate pathways shared by both groups. D) GSEA analysis comparing the Hep0 vs Hep2 subset (top) and Hep0 vs Hep9 subset (bottom). The first circle represents enriched pathways, with colors indicating secondary classifications. The second circle shows the number of enriched genes and their *p*‐values. The third circle depicts the ratio and number of upregulated/downregulated genes. The fourth circle illustrates the enrichment scores of pathways, with outward direction indicating upregulation and inward direction indicating downregulation.

The multi‐pathway enrichment analysis of upregulated genes across Hep1–9 revealed distinct differences in pathway activation (**Figure** **3**A). Hep1 was primarily characterized by upregulation of “Cholesterol metabolism”, while Hep2 exhibited significant alterations in “Fatty acid metabolism”. Hep3 showed strong activation of the “PPAR pathway”, along with features related to bacterial infections, including “Shigellosis, Salmonella infection, Bacterial invasion of epithelial cells, and Pathogenic Escherichia coli infection”. Hep4 was similar to Hep3 but also demonstrated upregulation in “Endocytosis and Platelet activation”. Hep5 exhibited activation of the “Hippo pathway and Glycosaminoglycan biosynthesis”. Hep6 was marked by upregulation in “Motor proteins, Phagosome, and Cholinergic synapse” pathways. Hep7 displayed increased “Bile secretion”, activation of the *“*cAMP signaling pathway”, and dysregulated “folate metabolism”. Hep8, a more complex subtype, exhibited a combination of features from Hep1/2/3/4/7, indicating higher malignancy. Hep9 showed similarities to Hep8, with upregulation in “Oxidative phosphorylation” pathways, but lacked increased “Bile secretion and cAMP signaling pathway activation”. Additionally, gene intersection analysis of the top 20 DEGs across Hep1–9 revealed several HCC‐related genes that were consistently downregulated in more than three hepatocyte subtypes, including *CDA, DDI2, IFNLR1, MAN1C1*, *PEX14*, and *PHACTR4* (Figure 3B). Notably, genes such as *ERRFI1* ^[^ ^23^ ^]^ and *PER3* ^[^ ^24^ ^]^ were significantly downregulated in Hep1/3/4/6. While most of these genes have been previously implicated in HCC, their specific roles across different hepatocyte subtypes remain poorly understood. Further functional validation and comprehensive studies are required to clarify their contributions to HCC initiation, progression, and clinical consequence.

Carlessi et al.^[^ ^25^ ^]^ used snRNA‐seq in combination with two mouse models to characterize the cellular microenvironment of healthy and precancerous livers. They identified a disease‐associated hepatocytes (daHep) subtype whose proportion progressively increased with chronic liver disease progression. These cells, characterized by elevated *ANXA2* expression, have been proposed as potential biomarkers for predicting HCC risk. Our snRNA‐seq analysis identified Hep3/4/5/6 as transitional‐phase hepatocyte subtypes, prompting us to investigate their potential connection to daHep. Further analysis of the gene expression profiles of the 10 hepatocyte subtypes revealed that Hep5 exhibits the highest *ANXA2* expression, while Hep0 shows the lowest (Figure S3B, Supporting Information). Additionally, upregulation of “Endocytosis” pathways observed in daHep was also found in Hep4 (Figure 3A). Downregulated pathways in daHep, such as “Complement and coagulation cascades, Valine, leucine, and isoleucine degradation, Fatty acid metabolism, Peroxisome and PPAR signaling pathway”, were also enriched in Hep3/4/5/6 (Figure S3A, Supporting Information). These findings strongly suggest that Hep3/4/5/6 represent transitional‐phase hepatocytes with potential roles in HCC progression, paralleling the characteristics of daHep.

Subsequently, we investigated the oncogenic landscape and molecular mechanisms associated with Hep0 compared to Hep2/9. Given the increased tumor heterogeneity in post‐cancerous hepatocytes, we defined Hep0 as a non‐cancerous state. Using the FindMarkers function in the Seurat package, we identified marker genes for Hep0 vs Hep2/9. Among the top 10 KEGG‐enriched pathways, five were commonly enriched in both Hep0 vs Hep2 and Hep0 vs Hep9 group (Table S7, Supporting Information): “Complement and coagulation cascades, Retinol metabolism, Peroxisome, Biosynthesis of amino acids, and Tryptophan metabolism” (Figure 3C). Downregulation of these pathways promotes liver cancer development,^[^ ^26^ ^]^ especially “Retinol metabolism and Tryptophan metabolism”, which were also validated in our bRNA‐seq data (Figure 1F). Similarly, comparisons of Hep9 vs Hep0 and Hep2 vs Hep0 revealed enrichment in tumor‐related pathways, including “Axon guidance,^[^ ^27^ ^]^ ECM‐receptor interaction,^[^ ^28^ ^]^ Chemical carcinogenesis reactive oxygen species,^[^ ^29^ ^]^ and Proteoglycans in cancer”^[^ ^30^ ^]^ (Figure S3C, Supporting Information). However, no shared pathways were found between these two groups, highlighting tumor cell heterogeneity in HCC.

Gene Set Enrichment Analysis (GSEA) further revealed changes in gene expression and their associated biological processes between the Hep0 vs Hep2 and Hep0 vs Hep9 groups (Figure 3D; Table S8 Supporting Information). Key metabolic pathways related to HCC, such as “Glycolysis/Gluconeogenesis, Carbon Metabolism, and Purine Metabolism,” were identified, providing insight into the biological mechanisms underlying HCC development and potential therapeutic targets.

### 2.4 Exploration of the Oncogenic Landscape of Hepatocytes by Integration of snRNA‐Seq with bRNA‐Seq

**Figure 4:**
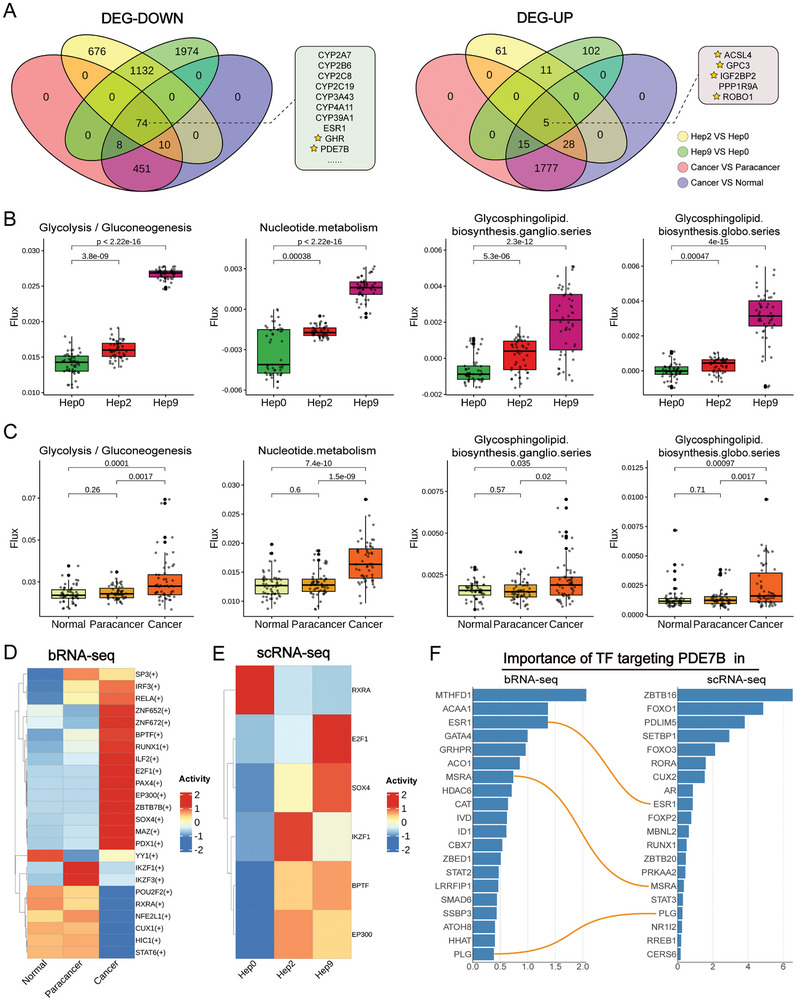
Exploration of the oncogenic landscape of hepatocytes by integration of bRNA‐seq with snRNA‐seq. A) Venn analysis of downregulated (left) and upregulated (right) DEGs. * indicate DEGs included in the QDM. B, C) Metabolic flux analysis derived from snRNA‐seq (B) and bRNA‐seq (C) data using METAFlux. D) Identification of differentially expressed regulators in various tissues from bRNA‐seq data using SCENIC. E) Identification of differentially expressed regulatory factors in hepatocyte subtypes from bRNA‐seq data using SCENIC. F) Identification of Transcription factors targeting *PDE7B* using bRNA‐seq and snRNA‐seq data. X‐axis represents the activity scores of the regulators.

To dissect the oncogenic regulatory mechanisms in HCC, we integrated snRNA‐seq and bRNA‐seq data from the SMU cohort, elucidated the molecular features driving the transition of hepatocytes to HCC. In snRNA‐seq, Hep0 was used as the baseline for identifying DEGs, whereas paracancer and normal tissues served as controls in bRNA‐seq. Venn analysis of these DEGs identified 74 commonly downregulated genes and 5 commonly upregulated genes relevant to HCC (**Figure** **4**A). Enrichment analysis using Metascape^[^ ^31^ ^]^ revealed that the commonly downregulated DEGs are associated with various chronic liver conditions, including “fatty liver disease, drug‐induced liver disease, and non‐alcoholic steatohepatitis.”

During HCC progression, we observed suppressed expression of multiple CYP family genes (e.g., *CYP2A7*, *CYP2B6*, *CYP2C8*, and *CYP2C19*) and significant perturbations in critical physiological metabolic processes such as “carboxylic acid metabolism, olefinic compound metabolism, and steroid metabolism.” Notably, we identified five significantly upregulated DEGs highly associated with HCC progression: *ACSL4*, *GPC3*, *IGF2BP2*, *PPP1R9A*, and *ROBO1*. All but *PPP1R9A* showed elevated expression in the TCGA cohort, suggesting their potential as biomarkers for HCC (Figure S4A, Supporting Information).

METAFlux, a computational framework capable of predicting cancer metabolic flux from bulk or single‐cell transcriptomic data, revealed metabolic heterogeneity and interactions within the tumor microenvironment.^[^ ^32^ ^]^ Using METAFlux, we assessed metabolic alterations across the Hep0/2/9 subtypes and identified progressive and significant differences in metabolic pathways including “glycolysis/gluconeogenesis, nucleotide metabolism, glycosphingolipid biosynthesis‐ganglio series, and glycosphingolipid biosynthesis‐globo series” (Figure 4B). In parallel, bRNA‐seq data showed significant upregulation of these four metabolic pathways in cancer tissues, suggesting their potential impact on HCC development and progression (Figure 4C; Figure S4C, Supporting Information).

The pathogenesis of HCC is linked to the aberrant activation of various oncogenes, with the regulatory network of transcription factors (TFs) offering a crucial avenue for exploring the disease's molecular mechanisms. By applying the SCENIC^[^ ^33^ ^]^ algorithm to bRNA‐seq data, we identified several upregulated regulons in cancer tissues, including *PDX1, MAZ*, and *SOX4* (Figure 4D). We then assessed regulon activity scores across Hep0/2/9 and compared them with regulators identified by bRNA‐seq. This analysis revealed five notable regulators: *E2F1*,^[^ ^34^ ^]^ *SOX4*,^[^ ^35^ ^]^ *BPTF*,^[^ ^36^ ^]^ *EP300*,^[^ ^37^ ^]^ *IKZF1*,^[^ ^38^ ^]^ and *RXRA* ^[^ ^39^ ^]^ (Figure 4E; Figure S4E, Supporting Information). While these regulators are known to be involved in liver cancer pathogenesis and progression, fully elucidating their regulatory targets remains elusive, highlighting the need for further detailed research.

Our study aimed to contribute to elucidating gene regulatory networks in HCC. Focusing on *PDE7B*, we utilized both bRNA‐seq and snRNA‐seq to predict transcription factors targeting *PDE7B*, ranking them by their importance coefficients (Figure 4F). From the intersection of these analyses, we identified three transcription factors as regulators of *PDE7B*: *ESR1*, *MSRA*, and *PLG*. Currently, there is a lack of studies examining the regulatory relationship between *PDE7B* and these transcription factors. Unraveling these regulatory mechanisms offers novel insights into the pathogenesis and progression of liver cancer.

### 2.5 Constructing the QDM Based on HCC Cohorts Referring snRNA‐Seq Findings

**Figure 5:**
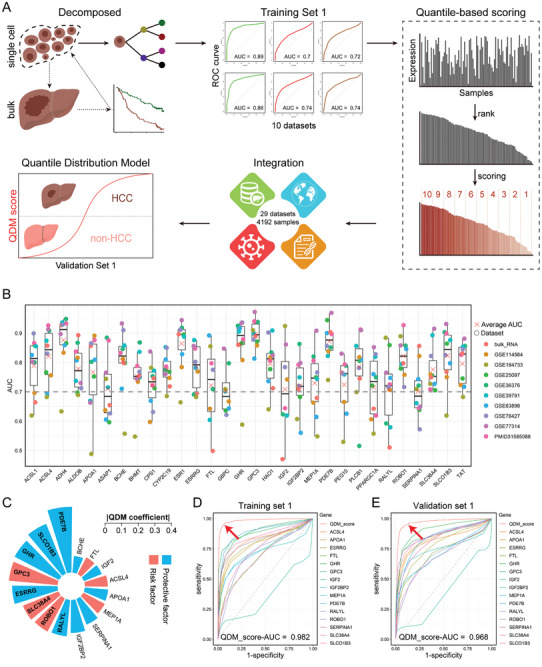
Constructing the QDM based on HCC cohorts referring snRNA‐seq findings. A) Workflow for constructing the QDM. B) AUC values for different genes in diagnosing liver cancer tissues across 10 datasets. C) Regression coefficients for each gene in QDM. D) ROC curves generated using the QDM for diagnosing HCC in Training Set 1. E) ROC curves generated using the QDM for diagnosing HCC in Validation Set 1.

To explore the potential clinical value of snRNA‐seq data, we integrated our snRNA‐seq results with public RNA‐seq data to construct a QDM applicable to HCC (**Figure** **5**A). We first decomposed^[^ ^40^ ^]^ the cellular composition of the TCGA HCC cohort (*n* = 371) based on the molecular profiles of 10 hepatocyte subtypes (Hep0–9) obtained from snRNA‐seq data (Figure S5A, Suppurting Information). Kaplan‐Meier survival analysis revealed that the patients with a higher proportion of the Hep0 subtype had significantly longer survival times (*p* = 0.0013), while those with a higher proportion of the Hep9 subtype had significantly shorter survival times (*p* = 0.045, Figure S5B, Supporting Information). Similarly, in the Fudan‐HCC cohort^[^ ^41^ ^]^ (*n* = 159, Figure S5A, Supporting Information), we observed a strong positive correlation between the proportion of the Hep0 subtype and survival time (*p* = 0.0011), as well as a strong negative correlation between the proportion of the Hep9 subtype and survival time (*p* = 0.0038, Figure S5B, Supporting Information). These findings align with our expectations, as Hep0 primarily represented non‐cancerous components from paracancer tissues, which may be relatively beneficial for long‐term survival, while Hep9 represented highly malignant subtype associated with poorer prognosis. Additionally, although Hep2 showed only a weak negative trend with survival time in the TCGA cohort, it demonstrated a strong negative correlation with survival time in the Fudan cohort (*p* < 0.0001).

Next, we employed mlr3verse^[^ ^42^ ^]^ machine learning R package to extract Hep0, Hep2, and Hep9 subtypes from all hepatocytes (training samples:test samples = 8:2) using the Random Forest algorithm. In a fivefold cross‐validation, the average classification accuracies for Hep0, Hep2, and Hep9 were 89.59%, 93.43%, and 99.44%, respectively. Notably, several established tumor suppressor genes (e.g., *ESR1*,^[^ ^43^ ^]^ *GHR*,^[^ ^14^ ^]^ *ALDOB*,^[^ ^44^ ^]^ *BAAT*,^[^ ^45^ ^]^ and *ALDH1A2* ^[^ ^46^ ^]^) were among the top 10 ranked feature genes in Hep0 (Figure S5C; Table S9, Supporting Information). Conversely, Hep9 was enriched with known tumor‐promoting genes (e.g., *AFP*, *PEG10*,^[^ ^47^ ^]^ *GPC3*,^[^ ^48^ ^]^ *IGF2BP2*,^[^ ^49^ ^]^ and *ACSL4* ^[^ ^50^ ^]^). We extracted the top 30 feature genes from Hep0/2/9 (total = 90) and plotted the receiver operating characteristic (ROC) curves for HCC diagnosis across ten datasets. Genes with an average area under the curve (AUC) below 0.70 were filtered out, resulting in 29 high‐performance diagnostic genes used to construct the diagnostic model (Figure 5B).

During the construction of the QDM, we faced two main challenges. The first challenge was the selection of a method for model construction. Although machine learning algorithms often achieve high diagnostic accuracy, they require high quality data and have limited generalization ability to new data. Therefore, we opted for the logistic regression algorithm due to its simplicity, ease of application to new data, and clinical utility. The second challenge was data integration. Despite the availability of algorithms for data integration (e.g., ComBat‐seq,^[^ ^51^ ^]^ sva,^[^ ^52^ ^]^ Ratio‐G,^[^ ^53^ ^]^ etc.), they often failed to effectively eliminate differential effects across datasets (including variations in detection platforms, methods, genes, and operations). To address this, we proposed a quantile‐based scoring algorithm: 1) Within each dataset, all original expression values for each gene were ranked, and the decile of each gene's expression value was calculated. 2) Each original expression was scored based on the decile (1–10 points). 3) The scored values from different datasets were combined directly. As scoring was independently conducted within each dataset, it effectively reduced background differences among different datasets. Using this quantile scoring algorithm, we integrated 29 human HCC datasets (4192 samples, Table S10, Supporting Information) from various countries, detection methods, and instrument platforms, regardless of the presence or type of hepatitis virus infection.

Starting with 29 genes that showed high diagnostic performance, we constructed a model using 10 scoring datasets (Training Set 1, *n* = 2314). During this process, we eliminated genes without significant differences and ultimately developed a QDM containing 16 genes. In the QDM regression formula, we defined the event of HCC tissue as 1 and non‐HCC tissue as 0. The regression coefficients for each gene indicated whether a gene acts as a protective factor (regression coefficient < 0) or a risk factor (regression coefficient > 0) for HCC occurrence (Figure 5C): QDM_score = 5.5942620 ─ 0.3870143×*PDE7B* ─ 0.3409017×*GHR* + 0.3371936×*GPC3* ─ 0.3187214×*SLCO1B3* ─ 0.2603766×*ESRRG* + 0.2396385×*SLC38A4* + 0.2373530×*ROBO1* ─ 0.2361836×*RALYL* ─ 0.1768778×*IGF2BP2* ─ 0.1744291×*SERPINA1* ─ 0.1508238×*APOA1* + 0.1467488×*MEP1A* + 0.1377588×*ACSL4* + 0.1109942×*FTL* ─ 0.1078583×*IGF2*. The ROC analysis of Training Set 1 yielded an AUC of 0.982 (Figure 5D). When applied to the remaining 19 datasets (Validation Set 1, *n* = 1878), the QDM predicted HCC and non‐HCC tissues with an overall AUC of 0.968 (Figure 5E). Additionally, in the SMU cohort, the diagnostic AUC of QDM reached 0.9868. The QDM demonstrated exceptionally high diagnostic performance in both Training Set 1 and Validation Set 1 (total = 4192), supporting integrated, evaluated, and diagnosed cross‐dataset and cross‐platform applications.

### 2.6 Investigation of QDM for its Biological and Clinical Significance

**Figure 6:**
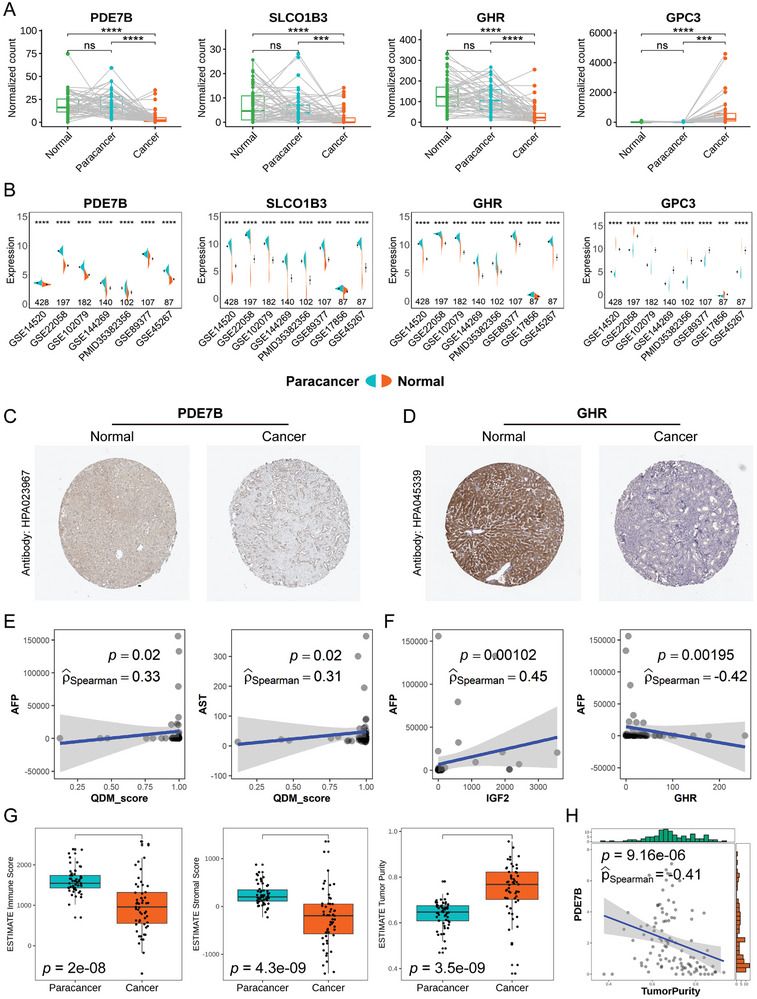
Investigation of QDM for its biological and clinical significance. A) The RNA expression levels of *PDE7B*, *GHR*, *GPC3*, and *SLCO1B3* in bRNA‐seq data from the SMU cohort were assessed and compared using paired *t*‐tests. ^ns^ *p* > 0.05; *p < 0.05; **p < 0.01; ***p < 0.001; ****p < 0.0001. B) The RNA expression levels of *PDE7B*, *GHR*, GPC3, and *SLCO1B3* were evaluated across different datasets using *t*‐tests. The number of samples is indicated above each dataset. ^ns^ *p*>0.05; *p < 0.05; **p < 0.01; ***p < 0.001; ****p < 0.0001. C, D) Protein expression levels of PDE7B (C) and GHR (D) were investigated using the HPA database. E) Correlation between QDM score and serum AFP and AST levels in the SMU cohort. F) Correlation of *IGF2* and *GHR* with serum AFP levels in the SMU cohort. G) Assessment of the tumor microenvironment in bRNA‐seq data from the SMU cohort using ESTIMATE to separately evaluate immune scores, stromal scores, and tumor purity. H) Correlation between *PDE7B* and tumor purity in the SMU cohort.

The QDM demonstrated robust predictive performance in diagnosing HCC tissues and provided valuable insights into the molecular mechanisms underlying HCC development, highlighting its significant impact on both scientific research and clinical practice. The 16 diagnostic genes identified by the QDM have been largely validated as crucial to HCC pathogenesis, exhibiting either tumor‐suppressive or oncogenic properties. For example, QDM identified protective factors such as *GHR*,^[^ ^14^ ^]^ *SLCO1B3*,^[^ ^54^ ^]^ and *APOA1*,^[^ ^55^ ^]^ alongside risk factors including *GPC3*,^[^ ^48^ ^]^ *SLC38A4*,^[^ ^56^ ^]^ and *ROBO1*.^[^ ^57^ ^]^ We analyzed the expression patterns of the top four genes (*PDE7B*, *GHR*, *GPC3*, and *SLCO1B3*) based on the absolute values of their regression coefficients. In the bRNA‐seq data from our SMU cohort, these genes exhibited markedly different expression levels in cancer tissues compared to paracancer or normal tissues (**Figure** **6**A). We further examined their expression in eight large‐scale HCC datasets (GSE14520, GSE22058, GSE102079, GSE144269, PMID35382356, GSE89377, GSE17856, and GSE45267) and found significant difference in cancer tissues vs their controls (Figure 6B). Additionally, the upregulation or downregulation direction matched the regression coefficients. The protein‐level expression of *PDE7B* and *GHR*, validated using the Human Protein Atlas (HPA) database,^[^ ^58^ ^]^ confirmed their downregulation in HCC cancer tissues compared to normal tissues (Figure 6C).

To investigate the clinical significance of the QDM, we collected clinical assays from the SMU cohort, including AFP and AST (indicator of liver function impairment). We observed a weak correlation between the QDM score and serum AFP levels (Spearman's coefficient = 0.33, *p* = 0.02) and serum AST levels (Spearman's coefficient = 0.3, *p* = 0.02) (Figure 6E). The Spearman's coefficient between *IGF2* (a diagnostic gene in QDM) and AFP was 0.45 (*p* = 0.00102), while for *GHR* (another diagnostic gene in QDM) and AFP, it was ─ 0.42 (*p* = 0.00195), suggesting potential for developing clinical biomarkers through QDM (Figure 6F). Using the ESTIMATE^[^ ^59^ ^]^ algorithm, we assessed the tumor microenvironment in bRNA‐seq data from the SMU cohort and found lower immune and stromal scores, and higher tumor purity in cancer tissues compared to paracancer tissues (Figure 6G). Furthermore, we identified a negative correlation between *PDE7B* and tumor purity (Spearman's coefficient = ─ 0.41, *p* <0.0001) (Figure 6H), which was highly expressed in Hep0.

To validate the reliability and comprehensibility of QDM, we investigated the tumor biological behavior of *PDE7B* in HCC cells. *PDE7B*, a member of the phosphodiesterase family, regulates various physiological processes by hydrolyzing cAMP and cGMP, including cell proliferation, differentiation, inflammation, and metabolic functions.^[^ ^60^ ^]^ It is implicated in tumor development of colorectal cancer,^[^ ^61^ ^]^ leukemia,^[^ ^62^ ^]^ and breast cancer;^[^ ^63^ ^]^ however, its specific role in HCC pathogenesis requires further investigation. Initially, we analyzed the gene expression of *PDE7B* in four HCC cell lines (Hep G2, PLC/PRF/5, MHCC‐97H, and SNU‐387) and found that *PDE7B* expression was highest in the SNU‐387 cell line (Figure S6A, Supporting Information), consistent with data from the HPA database (Figure S6B, Supporting Information). Therefore, we selected SNU‐387 cells for further experimental investigations. To assess the functional role of *PDE7B*, we designed two siRNA sequences (siPDE7B‐1 and siPDE7B‐2) and evaluated their interference efficiency using cell transfection and qRT‐PCR. The results indicated that the interference efficiency of siPDE7B‐1 was 70.0%, while siPDE7B‐2 showed an efficiency of 68.8% (Figure S6C, Supporting Infromation). Since no significant difference was observed between the two sequences, we selected siPDE7B‐1 (hereafter referred to as siPDE7B) for subsequent experiments. Following the transfection of SNU‐387 cells with siPDE7B, we performed a cell scratch assay 48 h post‐transfection and captured images at 0 and 24 h during the scratch assay. The results showed that the migration rate of the siPDE7B group was significantly higher than that of the control group (*p* <0.01, Figure S6D, Supporting Information). Additionally, CCK‐8 assays demonstrated that the cell viability of the siPDE7B group was notably higher than that of the control group (*p* <0.05, Figure S6E, Supporting Information). To summarize, inhibition of *PDE7B* significantly enhanced the migration ability and viability of HCC cells, which is consistent with the predictions made by the QDM. Future research will further explore the molecular mechanisms of *PDE7B* in HCC and assess its potential as a clinical biomarker for HCC.

Overall, the QDM demonstrated strong diagnostic performance and a significant correlation with clinical indicators. Most importantly, it holds great potential for identifying novel biomarkers with clinical relevance.

### 2.7 Classification of HCC Using Feature Genes Identified in snRNA‐Seq

**Figure 7:**
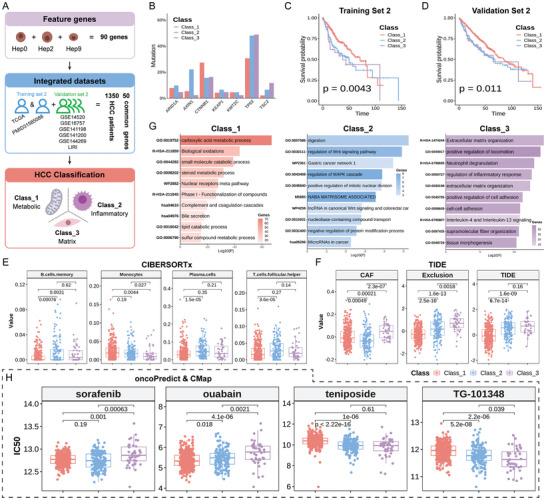
Classification of using feature genes identified in snRNA‐seq. A) Workflow for HCC classification using feature genes from snRNA‐seq data. B) Mutation frequencies of genes in Training Set 2. C, D) Kaplan‐Meier survival analysis for Training Set 2 (C) and Validation Set 2 (D). E) Immune cell composition in Validation Set 2. F) TIDE analysis for Training Set 2. G) Enrichment analysis of DEGs in Class_1, Class_2, and Class_3. H) Identification of sensitive drugs for Class_1, Class_2, and Class_3 through integration of OncoPredict and CMap analyses.

To improve the current classification of HCC in clinics, benefiting from the advanced scRNA‐seq message, we extracted the molecular features of the three distinct hepatocyte subtypes (Hep0/2/9) (**Figure** **7**A). We began by extracting feature genes from the snRNA‐seq data, selecting 30 genes for each subtype, resulting in a total of 90 genes. We then compiled eight human HCC datasets (*n* = 1350, Table S11, Supporting Information), which were processed to remove batch effects and divided into Training Set 2 and Validation Set 2. After excluding non‐expressed genes, we retained 50 common genes. Applying unsupervised consensus clustering to Training Set 2, we identified three distinct HCC classes: Class_1, Class_2, and Class_3.

Genomic studies have revealed that *CTNNB1* mutations are linked to well‐differentiated HCC tumors and extended overall survival,^[^ ^64^ ^]^ *AXIN1* mutations are associated with Wnt/β‐catenin pathway activation,^[^ ^65^ ^]^ frequent *TP53* mutations are correlated with poor prognosis,^[^ ^66^ ^]^ and *TSC2* mutations are often connected with significant matrix fibrosis and sclerosis in HCC.^[^ ^67^ ^]^ We examined gene mutation frequencies across the three HCC classes and found that Class_1 exhibited a significantly higher frequency of *CTNNB1* mutations compared to Class_2 and Class_3 (27.3%, 15.6%, and 16.3%, respectively) (Figure 7B). Conversely, the frequency of *TP53* mutations was notably lower in Class_1 (30.6%, 48.1%, and 48.8%, respectively), suggesting a lower risk of adverse prognosis for Class_1. Class_2 showed a higher frequency of *AXIN1* mutations (5.4%, 22.0%, and 2.3%, respectively), while Class_3 had a higher frequency of *TSC2* mutations (2.1%, 6.5%, and 11.6%, respectively).

To further investigate the clinical significance of these three classes, we performed survival analysis using integrated datasets (TCGA and PMID31585088) and observed that Class_1 exhibitedsuperior clinical outcomes (Figure 7C). Using TCGA and PMID31585088 as Training Set 2, we developed an HCC classification model with an accuracy of 91.58%. This model was then applied to Validation Set 2, confirming a significantly longer survival time in Class_1 compared to Class_2 and Class_3 (Figure 7D). CIBERSORTx analysis of the immune cell composition in Training Set 2 revealed that Class_2 exhibited a significantly higher abundance of various immune cell types (memory B cells, monocytes, plasma cells, and follicular helper T cells) compared to Class_1 and Class_3 (Figure 7E). In addition, TIDE^[^ ^68^ ^]^ analysis indicated that Class_3 exhibited higher levels of cancer‐associated fibroblasts (CAF) and T cell exclusion scores (exclusion) compared to Class_1 and Class_2, suggesting a highly immunosuppressive tumor microenvironment in Class_3 (Figure 7F).

DEGs analysis using Training Set 2 identified 164, 68, and 327 upregulated genes in Class_1, Class_2, and Class_3, respectively, with thresholds set at *p* < 0.05 and log2FoldChange > 1.5. Enrichment analysis revealed that Class_1 was predominantly associated with metabolic pathways, including “carboxylic acid metabolic process, biological oxidations, small molecule catabolic process, and steroid metabolic process” (Figure 7G). Class_2 was enriched in cancer‐related pathways, such as “regulation of Wnt signaling pathway, gastric cancer network 1, regulation of MAPK cascade, and lncRNA in canonical Wnt signaling and colorectal cancer” (Figure 7G). In contrast, Class_3 was associated with extracellular matrix‐related pathways, including “extracellular matrix organization, positive regulation of cell adhesion, and cell‐cell adhesion” (Figure 7G). Based on these findings, we classify Class_1, Class_2, and Class_3 as metabolic‐HCC, inflammatory‐HCC, and matrix‐HCC, respectively.

Classifying HCC enhances the optimization of individualized therapeutic strategies. We employed OncoPredict^[^ ^69^ ^]^ to assess the drug responsiveness of the three HCC classes to sorafenib and found that Class_3 exhibited a higher half‐maximal inhibitory concentration (IC50) compared to Class_1 and Class_2, indicating reduced sensitivity to sorafenib (Figure 7H). The Connectivity Map (CMap)^[^ ^70^ ^]^ is a gene expression‐based database designed to elucidate functional relationships between drugs and disease states. We conducted CMap analysis for each HCC class and integrating these findings with the OncoPredict, results identified the most sensitive drugs for Class_1, Class_2, and Class_3 as ouabain, teniposide, and TG‐101348, respectively. All three drugs demonstrated lower IC50 values than sorafenib, making them suitable for combination dosing regimens in the precision treatment of HCC.

## 3 Discussion

This study recruited an HCC cohort (SMU cohort) comprising 209 samples from 77 patients, and integrated snRNA‐seq and bRNA‐seq data to elucidate the oncogenic landscape of hepatocytes across subtypes, functional roles, and molecular regulations. We established the largest HCC snRNA‐seq dataset (174 756 cells) to date, providing valuable insights into hepatocyte oncogenesis. Using single‐cell results as a reference, we developed a quantile‐based scoring method to integrate 29 human HCC datasets from diverse sources, including different countries, hepatitis virus infections, detection methods, and instrument platforms. Based on this scoring method, we constructed a diagnostic model for HCC, termed QDM, which demonstrated excellent diagnostic performance across 4 192 samples (AUC = 0.982 for Training Set 1 and AUC = 0.968 for Validation Set 1). Notably, QDM not only correlates closely with clinical indicators but also enhances our understanding of HCC pathogenesis and facilitates the exploration of potential biomarkers. Through QDM, we identified numerous genes specifically associated with HCC, which could be useful for developing novel diagnostic biomarker combinations. The newly discovered genes warrant further investigation into their roles and mechanisms in HCC. Additionally, we identified several key metabolic pathways (including “glycolysis/gluconeogenesis, nucleotide metabolism, glycosphingolipid biosynthesis‐ganglio series, and glycosphingolipid biosynthesis‐globo series”) and transcription factors (particularly *E2F1*, *SOX4*, *BPTF*, *EP300*, *IKZF1*, and *RXRA*), providing important directions for HCC research. Based on the characteristics of snRNA‐seq data combined with multicenter cohorts, we classified HCC into metabolic‐HCC, inflammatory‐HCC, and matrix‐HCC classes and identified potential therapeutic agents for each class. This classification aims to enhance diagnostic accuracy, optimize personalized treatment strategies, and potentially uncover novel biomarkers or therapeutic targets, thereby improving patient prognosis and treatment outcomes. Future studies will further evaluate the robustness and reliability of this classification, explore the biological characteristics and molecular mechanisms of each class, assess their impact on therapeutic responses, and validate its clinical applicability.

Currently available transcriptomic datasets are primarily derived from microarray and RNA sequencing technologies, which differ significantly in detection principles, platforms, target genes, and data formats, complicating direct integration. In contrast, QDM effectively overcomes these obstacles by employing a quantile‐based scoring method, which converts gene expression values into relative expression values based on their rankings. This approach improves the robustness of the model, being interpretable, versatile, and iteratively extendable. We envision its clinical adoption to establish a multicenter molecular diagnostic system for QDM. Offering exceptional value as stated above, QDM has limitations. It requires a balanced number of HCC and non‐HCC samples to avoid skewed score distributions and necessitates complete gene expression data; while minor missing data can be managed through imputation methods, significant data loss remains problematic. The primary non‐HCC samples used in QDM are adjacent non‐tumorous tissues, while the discriminatory power may be further elevated when healthy liver tissues are applied as references. Moreover, large‐scale serum testing of diagnostic genes identified by QDM or with independent serum factors could further improve its predictive performance and clinical applicability for early screening, precision therapy, and dynamic monitoring of HCC.

Our data reveals the heterogeneity among HCC patients and cells, as well as alterations in abnormal metabolic pathways and the overexpression of transcription factors linked to HCC. Clearly, a single‐gene analysis is insufficient for deciphering the molecular networks of HCC. In future research, we aim to investigate the multifactorial mechanisms underlying HCC and to establish classification criteria based on metabolic pathways and transcription factors. To facilitate a user‐friendly diagnostic approach, we have focused on the top 30 genes from each hepatocyte subtype in the QDM. Expanding this gene list may help uncover additional diagnostic markers linked to HCC development amidst complex contributing factors, thereby advancing our understanding of the mechanisms of carcinogenesis.

In conclusion, this study elucidates the antagonistic roles of distinct hepatocyte subtypes in HCC and their associations with clinical indicators. By leveraging the characteristics of these subtypes, we have developed a high‐performance diagnostic model for HCC using multicenter datasets and identified potential biomarkers linked to the disease. This research offers the most comprehensive transcriptomic resource for human liver cancer to date and establishes a classification system based on hepatocyte subtypes. These advancements provide valuable insights for further exploration of hepatocarcinogenesis mechanisms and the development of therapeutic strategies for liver cancer.

## 4 Experimental Section

### Patient and Sample Collection

The clinical study was approved by the Medical Ethics Committee of Zhujiang Hospital, Southern Medical University (SMU), and informed consent was obtained from all participants. All research was conducted in accordance with the Declaration of Helsinki and the Declaration of Istanbul. The SMU cohort for this study comprised 77 patients who underwent HCC surgery at Zhujiang Hospital, Southern Medical University, between March 2019 and August 2023. A total of 209 paired samples, including cancer, paracancer, and normal tissues, were collected and preserved in liquid nitrogen for transcriptomic analysis. Additionally, portions of the tissue samples were fixed in 10% formalin solution and pathologically confirmed to contain HCC.

### Single Nucleus Extraction and snRNA‐Seq

The nuclei extraction reagents used in this study were prepared according to the 10x Genomics technical documentation (https://www.10xgenomics.com/support/single‐cell‐gene‐expression/documentation/steps/sample‐prep/nuclei‐isolation‐from‐adult‐mouse‐brain‐tissue‐for‐single‐cell‐rna‐sequencing), with subsequent optimization of the method. Briefly, liver tissue was minced into small pieces with a surgical blade, and 100 µL of cold 0.1X Lysis Buffer was added, then these were gently pipetted. The mixture was incubated on ice for 5 min. Next, 1 mL of cold Wash Buffer was added, thoroughly mixed, and the solution was filtered through a 40 µm cell filter to remove debris and large tissue fragments. The filtrate was centrifuged at 500 rcf for 5 min at 4 °C, and the supernatant was discarded without disturbing the nuclei pellet. The pellet was resuspended in 1 mL of cold Wash Buffer, gently mixed, and centrifuged again. This wash step was repeated twice. Finally, the nuclei pellet was resuspended in chilled Diluted Nuclei Buffer, and the nuclei concentration was determined using a Countess II FL Automated Cell Counter. The suspension was adjusted to the appropriate concentration, and single‐cell 3′ libraries were prepared using the 10x Genomics Single Cell 3′ Reagent Kit (v3) according to the manufacturer's protocol, followed by sequencing on an Illumina Novaseq X platform.

### Processing of the snRNA‐seq Data

The snRNA‐seq data were preprocessed, aligned to the human reference genome (GRCh38), and used to generate the raw gene expression count matrix using the Cell Ranger (version 6.0.1) pipeline. The Scrublet^[^ ^71^ ^]^ package was employed with default parameters to compute and remove doublets. Quality control was conducted using R software (version 4.0.4) and the Seurat package (version 4.0.5), retaining cells with mitochondrial UMIs < 10%, RNA counts > 200, and RNA features between 200 and 6 000. The data were scaled and subjected to dimensionality reduction using functions such as NormalizeData, FindVariableFeatures, ScaleData, and RunPCA within the Seurat package. Integration was further enhanced with the Harmony package, followed by non‐linear dimension reduction using the RunUMAP function and clustering analysis with the FindNeighbors and FindClusters functions. Automated annotation of cell clusters was performed using the ScType^[^ ^72^ ^]^ package, with subsequent manual refinement based on established marker genes. The code used in this study was accessible on the tutorial website (https://github.com/suhuanhou/2024_HCC), which included detailed documentation of the versions for each analytical environment and package.

### Procedure for bRNA‐Seq

Total RNA was extracted from tissues using the RNAiso Plus reagent (Takara, Japan), and RNA quantification was performed using a NanoDrop 2 000 spectrophotometer (Thermo Scientific, USA). The cDNA libraries were constructed using the SEQUMED MustSeq 3′ mRNA DEG kit (Sequmed, China). The prepared libraries were sequenced on the Illumina NovaSeq 6 000 platform, generating 150 bp paired‐end reads.

### Analysis of the bRNA‐seq Data

Quality control of raw sequencing reads was performed using Trimmomatic,^[^ ^73^ ^]^ removing adapters and filtering out low‐quality data to obtain clean reads. The reads were aligned to the human reference genome (GRCh38) using HISAT2,^[^ ^74^ ^]^ and gene expression levels were quantified as read counts based on the alignment results, followed by the calculation of reads per million mapped reads (RPM). Read count normalization was conducted using DESeq2, with differential expression analysis applying thresholds of log2FoldChange > 1.5 and *p* < 0.05. Additionally, RPM data were used for analyses with specific algorithms such as ESTIMATE and METAFlux. Detailed procedures were provided on the tutorial website (https://www.github.com/suhuanhou/2024_HCC).

### Developing a QDM for the HCC Tissues

We collected 29 human HCC datasets (total samples = 4 192), which included both HCC and non‐HCC samples. These datasets comprised 19 microarray datasets and 10 RNA‐seq datasets, with RNA‐seq data converted from FPKM or RPKM to TPM or CPM. A quantile‐based scoring algorithm was developed to integrate these diverse datasets across different detection platforms, methods, genes, and operations. Hepatocyte subtypes associated with patient survival in the snRNA‐seq data were identified, and machine learning methods were used to extract their feature genes. Initially, these feature genes were filtered across 10 training datasets, resulting in 30 genes with an AUC > 0.7 for diagnosing HCC. A logistic regression model, including 16 diagnostic genes, was constructed and optimized using the training datasets and validated in independent validation datasets.

### Cell Culture

The human HCC cell lines used in this study were obtained from Biospecies (Guangzhou, China) and authenticated by Short Tandem Repeat (STR) profiling to confirm their authenticity. All cell lines tested negative for mycoplasma, bacterial, yeast, and fungal contamination. Cells were cultured under optimal growth conditions at 37 °C in a 5% CO_2_ incubator. Specifically, Hep G2 and PLC/PRF/5 cell lines were maintained in MEM medium (Gibco, USA) with 10% fetal bovine serum (FBS, Gibco, USA). MHCC‐97H cells were cultured in DMEM medium (Gibco, USA) with 10% FBS, while SNU‐387 cells were cultured in RPMI‐1640 medium (Gibco, USA), supplemented with 10% FBS, 1% L‐glutamine (Biospecies, Guangzhou, China), and 1% sodium pyruvate (Biospecies, Guangzhou, China).

### Cell Transfection

Cells in the logarithmic growth phase at ≈ 80% confluency were harvested for siRNA transfection. To ensure experimental accuracy, all consumables were RNase‐free. Transfection was conducted using Lipofectamine 3 000 reagent (Thermo Scientific, USA) and Opti‐MEM medium (Gibco, USA), following the manufacturer's instructions. Six to eight hours of post‐transfection, the medium was replaced with fresh culture medium. The cells were cultured further or processed based on subsequent experimental requirements. The siRNAs used in this study were synthesized by Tsingke (China) and included the following: siRNA negative control (siNeg), siPDE7B‐1 (sense: 5′‐CACCAUUUCAAGUUAGAUA(dT)(dT), antisense: 5′‐UAUCUAACUUGAAAUGGUG(dT)(dT)), and siPDE7B‐2 (sense: 5′‐CGCCUACUUAACAGUACAA(dT)(dT), antisense: 5′‐UUGUACUGUUAAGUAGGCG(dT)(dT)).

### RNA Extraction and qRT‐PCR Detection

Total RNA was extracted from cells using the RNAiso Plus reagent (Takara, Japan), and RNA quantification was performed with a NanoDrop 2 000 spectrophotometer. mRNA expression was detected using HiScript II Q RT SuperMix (Vazyme, China) for reverse transcription and SYBR qPCR Master Mix (Vazyme, China) for quantitative PCR. Primers were synthesized by Sangon Biotech (China), with ACTB used as the internal control. The primer sequences were as follows: PDE7B (forward: 5′‐TTGACTTCCGCCTACTTAACAGT‐3′, reverse: 5′‐TAATTCCACGAAGCAGCCTTG‐3′). The relative quantification method (2^‐ΔCt) was used to calculate fold changes in gene expression.

### Cell Scratch Assay

Confluent monolayer cells in a 6‐well plate were scratched with a sterile 10 µL pipette tip. Afterward, the cells were washed twice with PBS to remove debris and cultured in medium without FBS. Images of the scratch were captured at 0 and 24 h, and the scratch width was measured using ImageJ software (version 1.52a) to calculate the rate of cell migration.

### CCK‐8 Assay

Approximately 1 000 cells were seeded in a 96‐well plate, with five replicates for each group. After overnight culture, siRNA transfection was performed. Six to eight hours of post‐transfection, the medium was replaced with fresh medium. After an additional 48 h, the cells were washed with PBS, followed by the addition of 90 µL of fresh medium and 10 µL of CCK‐8 reagent. The cells were incubated for another 4 h, and absorbance at 450 nm was measured. Cell viability was calculated using the following formula: Cell viability (%) = [(OD_experimental group – OD_blank) / (OD_control group – OD_blank)] × 100%.

### Statistical Analysis

All statistical analyses were performed using R (version 4.2.3) or GraphPad Prism (version 9.0.0). Unless otherwise specified, data are presented as mean ± SEM or individual data points. The Wilcoxon rank‐sum test for multiple comparisons was performed using the stat_compare_means function from the ggplot2 package. Kaplan‐Meier survival curves were visualized using the survival package, with Log‐rank tests conducted using the survdiff function. Statistical significance was indicated by asterisks: ^ns^ *p* > 0.05; *p < 0.05; **p < 0.01; ***p < 0.001; ****p < 0.0001.

## Conflict of Interest

The authors declare no conflict of interest.

## Author Contributions

H.S., X.Z., G.L., and C.L. contributed equally to this work. H.S. conducted bioinformatics analysis and drafted the manuscript. X.Z., C.L., and Y.C. handled sample processing and sequencing. C.L., J.Y., and C.X. managed clinical sample collection and patient information. Z.W., J.L., X.L., and Y.W. provided technical support. W.M. and G.L. contributed to data interpretation. X.L. and Y.C. were involved in manuscript revision and the result discussion. H.S., C.L., and P.K.H.T. assisted with manuscript review and provided clinical insights. X.P. conceived and designed the study, interpreted the result, and finalized the manuscript. P.K.H.T., C.L., H.S., and X. P. are co‐corresponding authors.

## Supporting information



Supporting Information

Supporting Information

## Data Availability

The bRNA‐seq and snRNA‐seq sequencing data are available in the Genome Sequence Archive (https://ngdc.cncb.ac.cn/gsa‐human/browse/HRA007785). The corresponding bRNA‐seq and snRNA‐seq expression data have been deposited in the OMIX,^[^ ^75^ ^]^ China National Center for Bioinformation/Beijing Institute of Genomics, Chinese Academy of Sciences (https://ngdc.cncb.ac.cn/omix, Accession: OMIX008374). Metadata for the bRNA‐seq and snRNA‐seq datasets are provided in Tables S1 and S4. Public datasets used in this study are detailed in supplementary table (Table S10 and 11). Analytical code employed in this study can be accessed on tutorial website (https://www.github.com/suhuanhou/2024_HCC). Additional data can be requested from the authors upon reasonable request.
