# Supplementary material for: Deciphering the Oncogenic Landscape of Hepatocytes Through Integrated Single‐Nucleus and Bulk RNA‐Seq of Hepatocellular Carcinoma
Source: Adv Sci (Weinh). 2025 Feb 17;12(14):2412944. doi: 10.1002/advs.202412944 (PMC11984907; doi:10.1002/advs.202412944)
Supplement: Supplementary file 1 — Supporting Information [file ADVS-12-2412944-s001.docx]

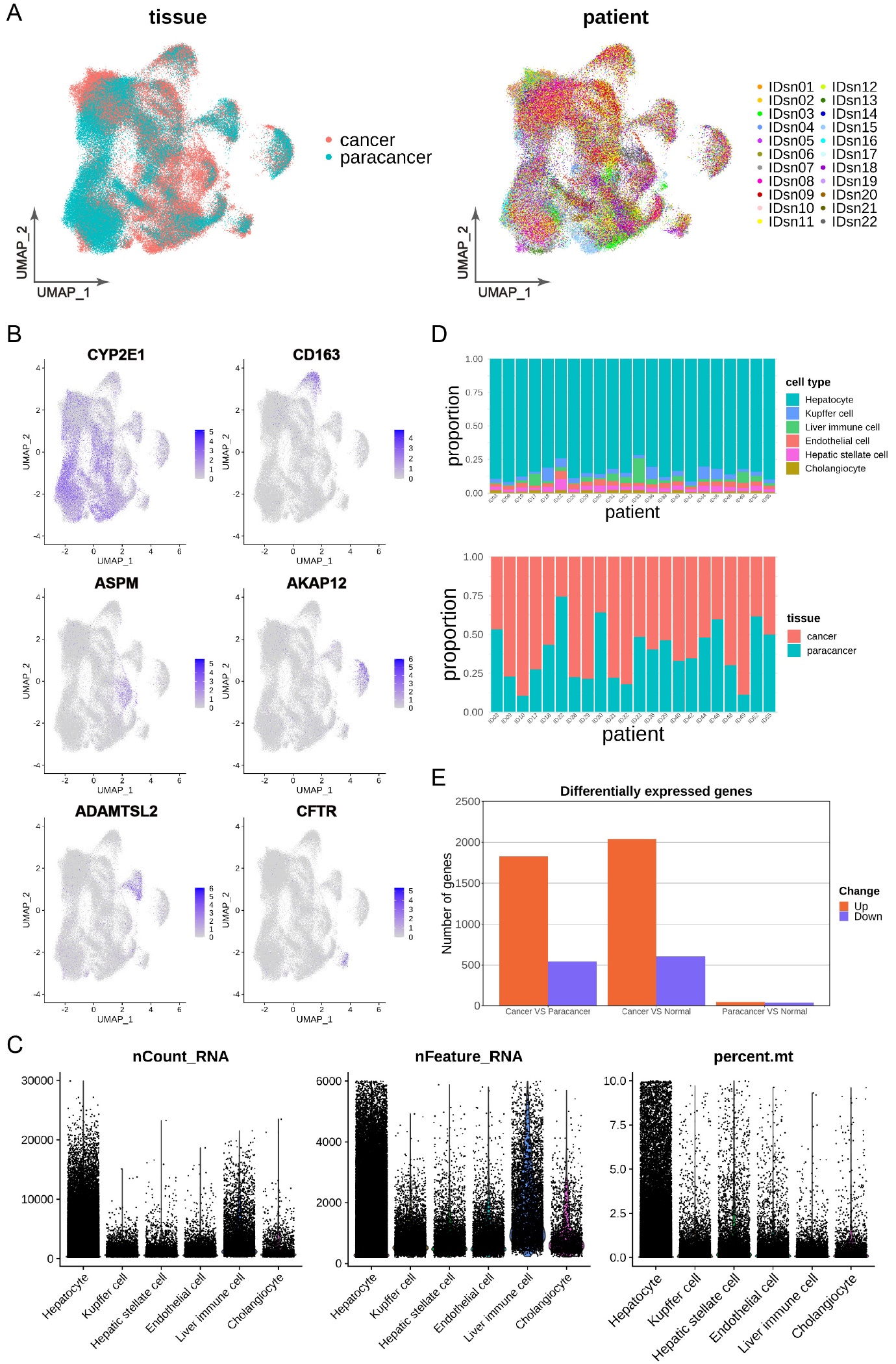


Figure S1 Cellular and gene composition of HCC based on bRNA-seq and snRNA-seq data.

(A) Distribution of tissue origins (left) and patient sources (right) in the snRNA-seq dataset.

(B) Expression patterns of marker genes across six cell types.

(C) Quality control metrics for snRNA-seq data, including RNA counts (left), RNA features (middle), and the proportion of mitochondrial genes (right).

(D) Cellular composition (top) and tissue origin (bottom) in the HCC snRNA-seq dataset

(E) Number of differentially expressed genes (DEGs) identified in the HCC bRNA-seq dataset.


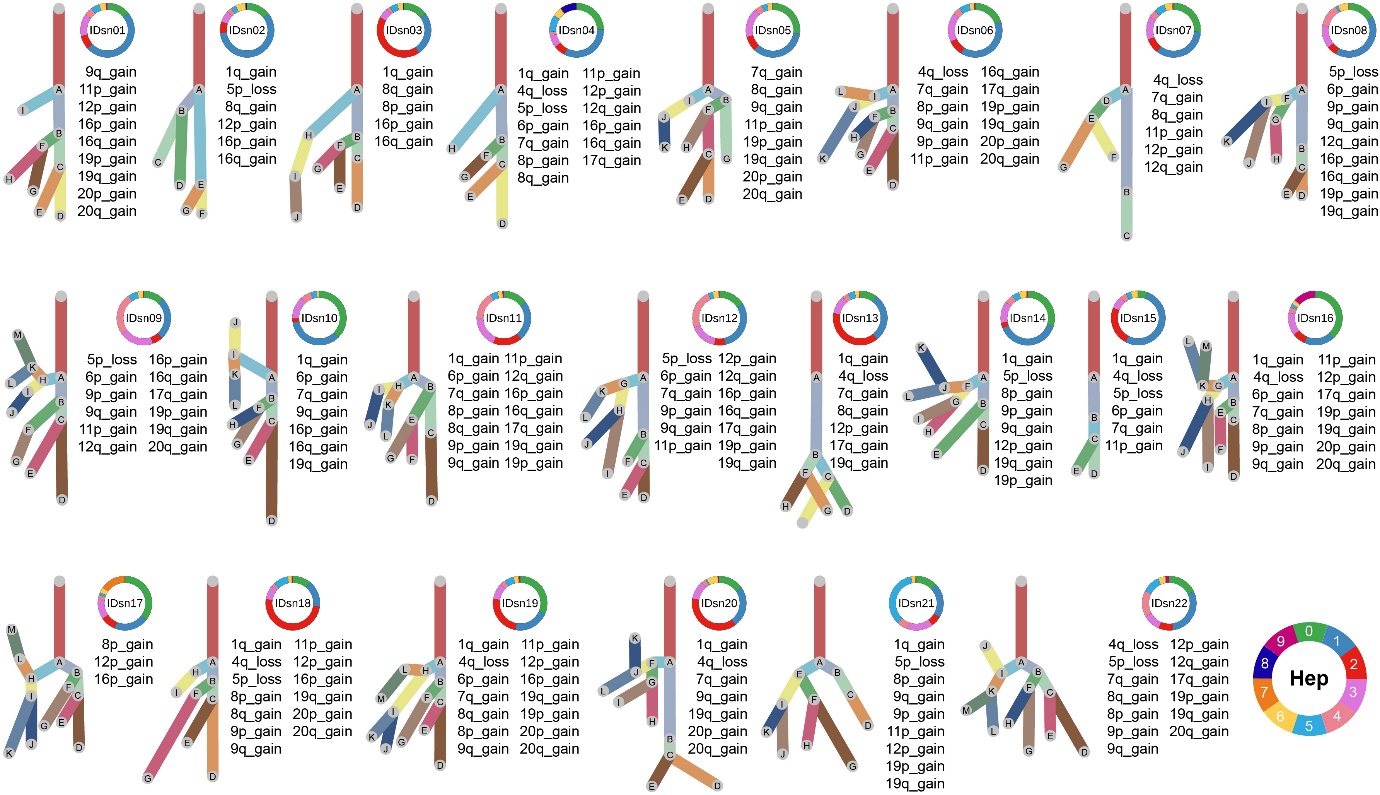


Figure S2 Clonality tree for each of the 22 HCC patients constructed using inferCNV analysis of snRNA-seq.

The labeled events represent early CNVs observed in more than 30% of patients. The accompanying donut plots display the hepatocyte subtype composition for each patient. Branch lengths, except for the root, are scaled to represent the proportion of cells with the corresponding CNVs in each subclonal population.


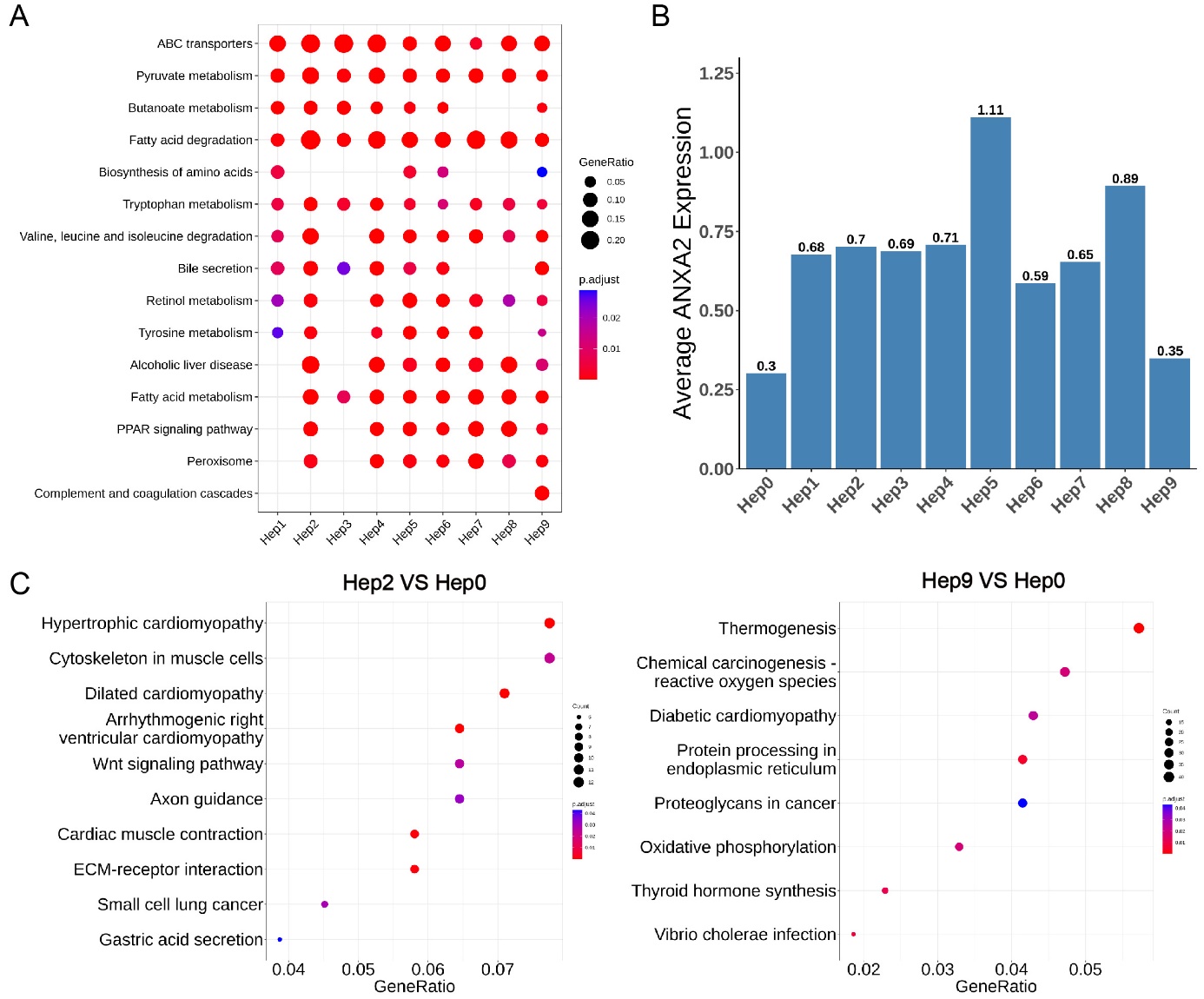


Figure S3 Enrichment analyses and gene expression profiles of hepatocyte subtypes.

(A) Multi-pathway enrichment analysis of downregulated genes across Hep1-9 subtypes.

(B) *ANXA2* expression across 10 hepatocyte subtypes.

(C) KEGG analysis of marker genes for Hep2 and Hep9 subtypes (left/right), using Hep0 as the control.


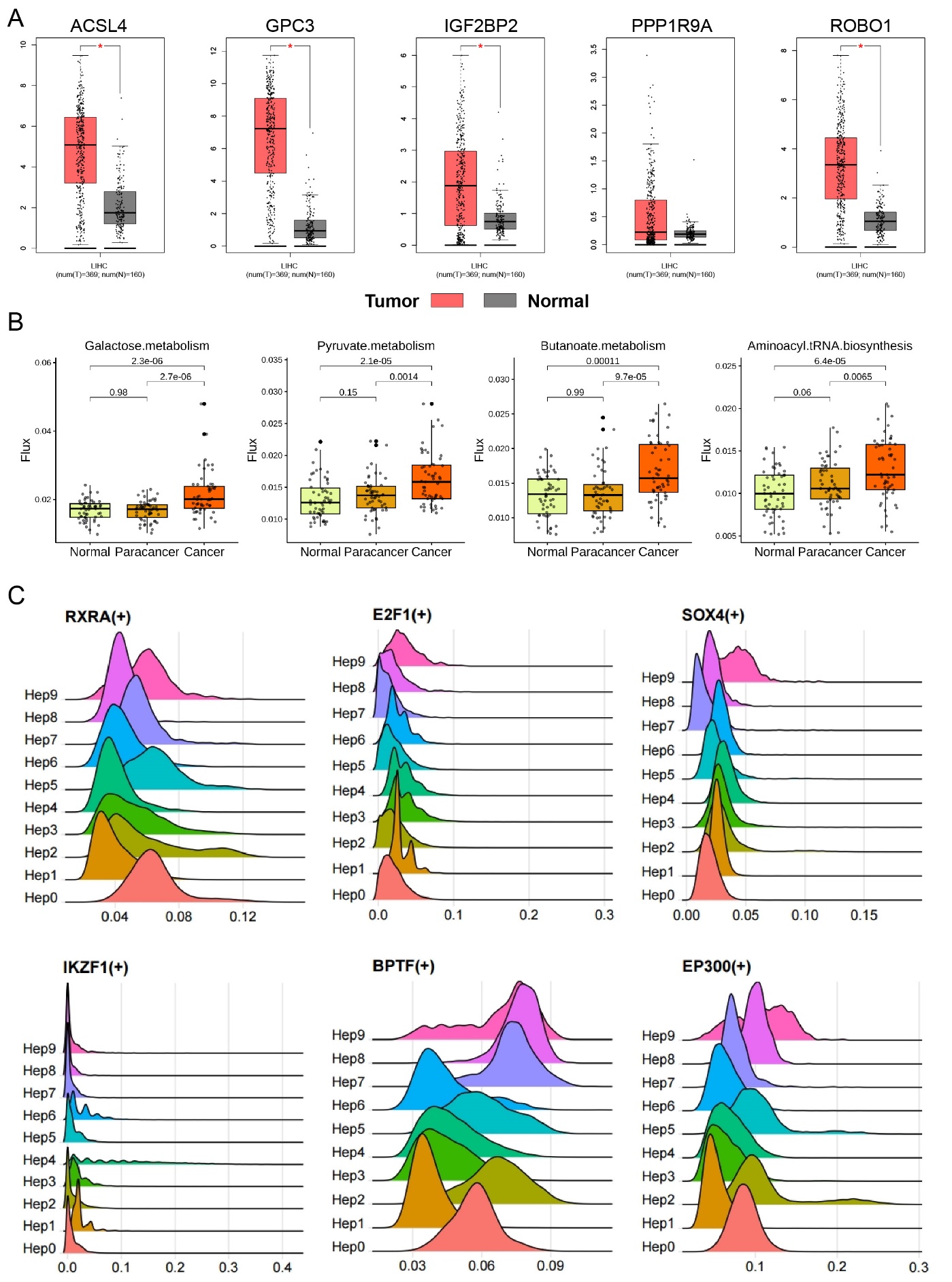


Figure S4 DEGs, metabolic pathways, and transcriptional regulators associated with HCC.

(A) Expression profiles of representative co-upregulated DEGs in the TCGA cohort.

(B) Metabolic pathways significantly upregulated in HCC tissues.

(C) Expression levels of transcriptional regulators across hepatocyte subtypes, with X-axis indicating regulator activity score, and Y-axis referring expression level.


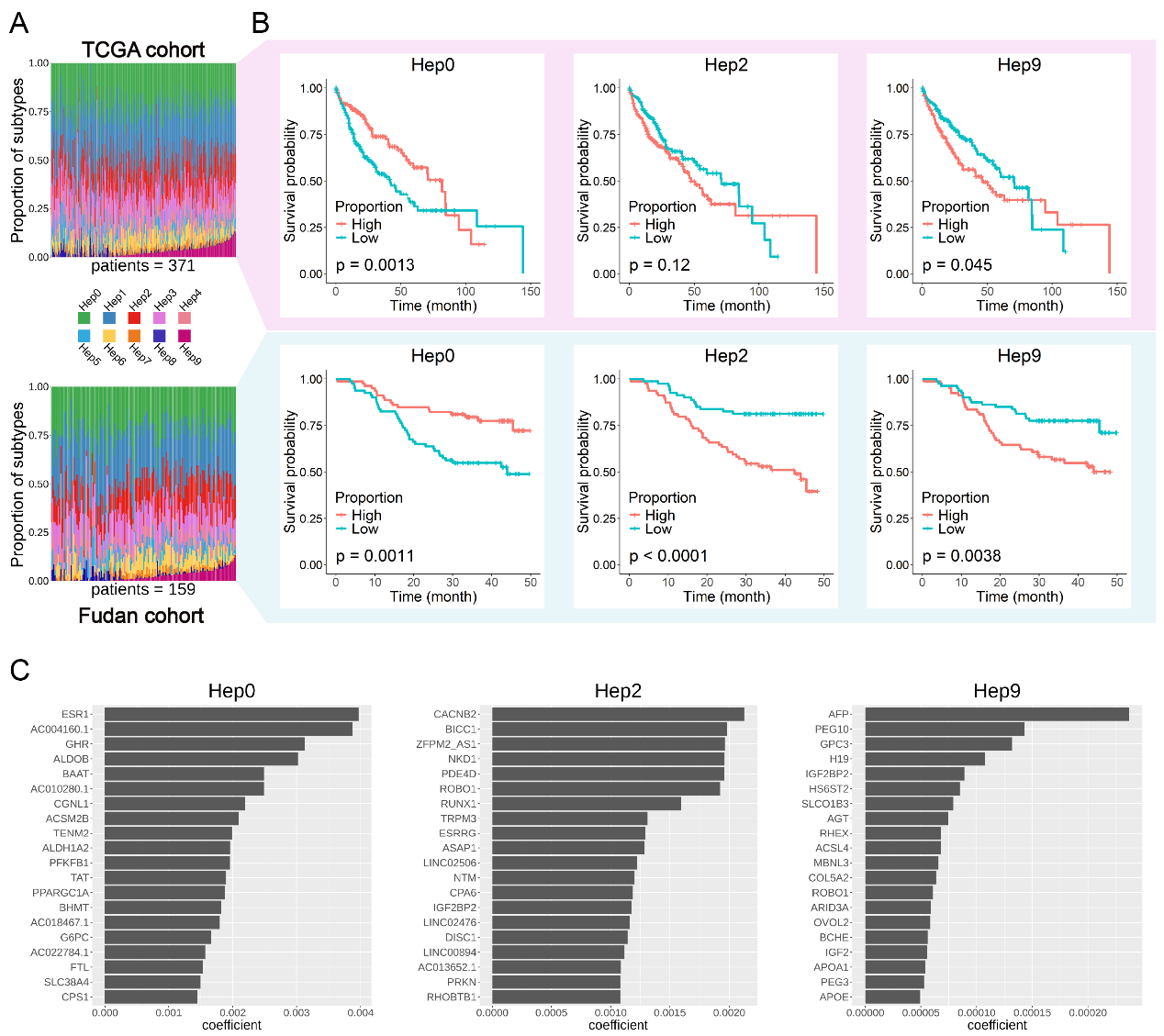


Figure S5 Deconvoluting of hepatocyte subtypes and survival analysis in two cohorts.

(A) Decomposition of hepatocyte subtypes using snRNA-seq data in the TCGA-LIHC and Fudan cohort.

(B) Correlation of Hep0/2/9 with survival outcomes.

(C) Top 10 ranked feature genes in Hep0/2/9, identified through training with mlr3verse.


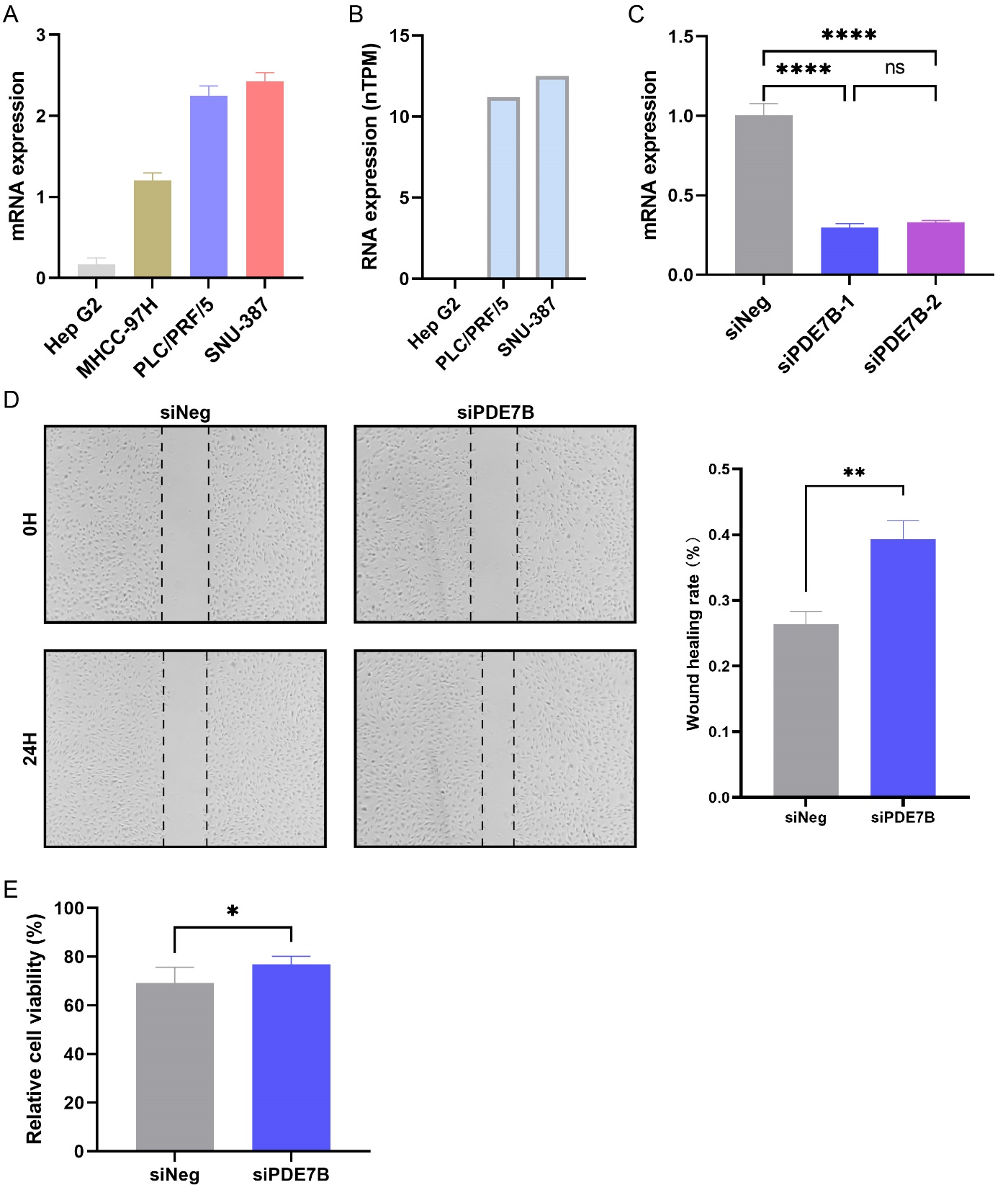


Figure S6 Functional characterization of *PDE7B* in SNU-387 cells.

(A) *PDE7B* expression levels across four HCC cell lines: Hep G2, PLC/PRF/5, MHCC-97H, and SNU-387).

(B) Validation of *PDE7B* expression in HCC cell lines using data from the HPA database.

(C) Interference efficiency of siPDE7B-1 and siPDE7B-2 in SNU-387 cells.

(D) Cell migration analysis of SNU-387 cells transfected with siPDE7B.

(E) Cell viability assay of SNU-387 cells transfected with siPDE7B. Remarks: ^ns^*p* > 0.05; **p* < 0.05; ***p* < 0.01; *****p* < 0.0001.
